# Scenario-Based Multi-Objective Optimum Allocation Model for Earthquake Emergency Shelters Using a Modified Particle Swarm Optimization Algorithm: A Case Study in Chaoyang District, Beijing, China

**DOI:** 10.1371/journal.pone.0144455

**Published:** 2015-12-07

**Authors:** Xiujuan Zhao, Wei Xu, Yunjia Ma, Fuyu Hu

**Affiliations:** 1 Key Laboratory of Environmental Change and Natural Disaster of Ministry of Education, Beijing Normal University, Beijing, China; 2 Academy of Disaster Reduction and Emergency Management, Ministry of Civil Affairs & Ministry of Education, Beijing Normal University, Beijing, China; Beihang University, CHINA

## Abstract

The correct location of earthquake emergency shelters and their allocation to residents can effectively reduce the number of casualties by providing safe havens and efficient evacuation routes during the chaotic period of the unfolding disaster. However, diverse and strict constraints and the discrete feasible domain of the required models make the problem of shelter location and allocation more difficult. A number of models have been developed to solve this problem, but there are still large differences between the models and the actual situation because the characteristics of the evacuees and the construction costs of the shelters have been excessively simplified. We report here the development of a multi-objective model for the allocation of residents to earthquake shelters by considering these factors using the Chaoyang district, Beijing, China as a case study. The two objectives of this model were to minimize the total weighted evacuation time from residential areas to a specified shelter and to minimize the total area of all the shelters. The two constraints were the shelter capacity and the service radius. Three scenarios were considered to estimate the number of people who would need to be evacuated. The particle swarm optimization algorithm was first modified by applying the von Neumann structure in former loops and global structure in later loops, and then used to solve this problem. The results show that increasing the shelter area can result in a large decrease in the total weighted evacuation time from scheme 1 to scheme 9 in scenario A, from scheme 1 to scheme 9 in scenario B, from scheme 1 to scheme 19 in scenario C. If the funding were not a limitation, then the final schemes of each scenario are the best solutions, otherwise the earlier schemes are more reasonable. The modified model proved to be useful for the optimization of shelter allocation, and the result can be used as a scientific reference for planning shelters in the Chaoyang district, Beijing.

## Introduction

Increasing attention is being paid to the impacts of natural disasters on humans as both society and technology develop [1,2]. Numerous retroactive and proactive countermeasures have been introduced to reduce the number of casualties and other impacts caused by natural hazards. The development of emergency shelters has been proved to be one of the most effective methods to reduce causalities, as they can provide safe havens for evacuees before, during and after a disaster [3].

The location and allocation of emergency shelters have long been critical and difficult issues. Many models have been developed based on research into site selection problems, including the p-median model [4], the p-center model [5] and covering models, such as the set covering model [6] and the maximal covering location model [7]. In terms of objectives, disaster shelter allocation models can be classified into two categories, namely, single-objective models and multi-objective models. Compared with multi-objective models, single-objective models (e.g. with the objective of minimizing travel distance or minimizing the construction cost) are easier to formulate and are well developed. For example, Sherali *et al*. [8] proposed a shelter model based on the p-median problem with the objective of minimizing the evacuation time to hurricane/flood emergency shelters. Based on a set covering model, Dalal *et al*. [9] studied the selection of sites for typhoon shelters in the countryside by adding a capacity constraint and a distance constraint. Their model was solved by applying a clustering analysis method. Based on the capacitated-median model, and considering the different function of relief points in various levels and the connection between them, Widener [10] and Widener and Horner [11] developed a hierarchical capacitated-median model and applied it to the location-allocation problem of hurricane relief points in Leon county, Florida, USA. Considering the hierarchical location problems of China, Chen and You [12] divided the facility system into two levels, a basic level and an advanced level, and built a hierarchical location model.

However, single-objective models are too simple to cover the complexity of the actual situation in the shelter location problem, which makes the development of multi-objective models necessary. Alçada Almeida *et al*. [13] built a shelter location model based on the p-median model with four objectives (minimizing the total distance to the shelter, minimizing the total risk of the primary path being impassable, minimizing the fire risk at the shelter and minimizing the total time from the shelter to the University Hospital) and solved this model within the framework of a web-based decision support system. Doerner *et al*. [14] proposed a location model for hurricane disaster facilities with the objectives of a weighted mean of a minimum and a maximum coverage criterion, minimizing the risk of tsunami events and minimizing costs. Adding the objectives of minimizing the construction cost of the facilities and the travel cost into a maximal covering location model, Barzinpour and Esmaeili [15] developed a multi-objective mixed-integer linear programming model by using a virtual zoning approach, achieving both the humanitarian and financial goals.

An optimum algorithm has been introduced to solve these models because the complexity of the location problem, which involves multiple objectives, strict constraints and a discrete feasible domain, makes it impossible to solve simply using GIS spatial analysis technology. For example, Li *et al*. [16] modified an ant colony algorithm, combined it with GIS technology, and applied it to a high-dimensional location problem. Hu *et al*. [17] modified the particle swarm optimization (PSO) algorithm and used it to solve location models for earthquake shelters. Yaghini *et al*. [18] developed a heuristic algorithm based on the local branching and relaxation-induced neighborhood search method and applied it to solving the capacitated p-median problem. Hu *et al*. [19] used a non-dominated sorting genetic algorithm to solve the multi-objective location model for earthquake shelters. Although there are various evolution algorithms to solve these models, the PSO algorithm has been proved to be simpler and more robust than other algorithms. However, as an evolutionary algorithm, the PSO algorithm cannot find the accurate solution in many problems and it is therefore necessary to modify this algorithm to find a more optimum solution.

The final objective in the facility location and allocation problem is to obtain the maximum profit with the lowest cost. For disaster emergency shelters, this final objective can be represented as the shortest evacuation time with the lowest construction cost for the shelter. Although there have been a number of studies on shelter planning, a more practical model is required as a result of the gaps between existing shelter models and real evacuation situations. The work reported here developed an earthquake shelter allocation model with the two objectives of minimizing the total shelter area and minimizing the total weighted evacuation time; the two constraints were the shelter capacity and the service area. The effects of hazards on the number of potential evacuees, as estimated by the building collapse rate, were also considered in three different scenarios using the Chaoyang district, Beijing, China as a case study. The evacuation speed of the residents was calculated by weighting the percentage of the population of different ages. To solve this complex problem, the PSO algorithm was modified by adding an outside loop and was combined with the simulated annealing (SA) algorithm to obtain a more optimum solution.

## Method

### Model formulation

For simplicity, the residents in a community were assumed to be concentrated at its center. It was also assumed that all the residents would evacuate to the same shelter at the same speed. It was also assumed that the construction cost of a shelter is determined by its size or capacity and therefore the objective of minimizing the construction cost could represented by minimizing the total capacity of the shelter.

Thus, a location model with two objectives of minimizing the total area of the shelters and minimizing the total weighted evacuation time, and with the constraints of the capacity of the shelters and the service distance, was proposed:

$$f_{1}=\text{min}\sum_{i=1}^{N}Y_{i}\times S_{i}$$ (1)

$$f_{2}=\text{min}\sum \frac{d_{ij}}{v_{j}}\times \frac{P_{j}}{W_{ji}}$$ (2)

Subject to

$$\sum_{j=1}^{M}P_{j}LB_{ji}-S_{i}Y_{i}\leq 0$$ (3)

$$C_{ji}B_{ji}-D_{j}\leq 0\;(\forall i=1,2,\ldots \ldots ,N\;\forall j=1,2,\ldots \ldots ,M)$$ (4)

$$\sum_{i=1}^{N}B_{ji}Y_{i}=1\;(\forall j=1,2,\ldots \ldots ,M)$$ (5)

$$B_{ji}\in (0,1)\;Y_{i}\in (0,1)$$ (6)

$$Y_{i}=\left\{ \begin{array}{l} 1,\;the\;candidate\;shelter\;i\;is\;selected\;as\;earthquake\;emergency\;shelter\;\\ 0,\;the\;candidate\;shelter\;i\;is\;not\;selected\;as\;earthquake\;emergency\;shelter\; \end{array}\right.$$ (7)

The variables, sets and parameter in the model are as follows:

*I* is the set of candidate facilities: *I* = (1, 2, … *i*, *… N)*
*J* is the set of the community: *J* = (1,2, … *j*, *… N)*
*S*_*i*_ is the area of the candidate shelter *i*
*L* is the smallest refuge area per capita (1 m^2^/person)
*d*_*ji*_ is the length of the shortest path between the community *j* and the candidate shelter *i*
*v*_*j*_ is the evacuation speed of the population group in the *j*th community
*D*_*j*_ is the maximum evacuation distance for the people in community *j*
*W*_*ji*_ is the mean width of the route from community *j* to candidate shelter *i*
*P*_*j*_ is the number of people who need to be evacuated in community *j*

Eq 1 represents the objective function *f*_1_ of minimizing the total area of the shelter and Eq 2 represents the objective function *f*_2_ of minimizing the total weighted evacuation time. The physical characteristics of the evacuees are one of the major factors affecting the evacuation speed. Assuming that the evacuation speed of the residents of a community depends on the age structure of the population in the community, and that each child needs the assistance of an adult during evacuation, then the evacuation speed *v*_*j*_ can be calculated according to Eq 8:

$$v_{j}=(2\times p_{c}\times v_{c}+(p_{a}-p_{c})\times v_{a}+p_{o}\times v_{o})\times \rho$$ (8)

where *v*_*c*_ is the speed of the child, *p*_*c*_ is the percentage of children in the community, *v*_*a*_ and *p*_*a*_ denote the speed and percentage of the adults in the community and *v*_*o*_ and *p*_*o*_ are the speed and percentage of elderly people in the community. The adjustment parameter ρ is set to 1.3 by considering that the evacuation speed is 1.3 times that in normal situations without considering the influence of collapsing buildings. The values of *v*_*c*_, *v*_*a*_ and *v*_*o*_ were decided by referring to the work of Gates *et al*. [20]. The population *P*_*j*_ in community *j* that needs to evacuate can be calculated according to Eq 9:

$$P_{j}=pop_{j}\times R$$ (9)

where *pop*_*j*_ is the population of community *j* and *R* is the evacuation rate for different scenarios.

Eq 3 denotes the capacity constraint that the area of the shelters should satisfy the demands of the residents. Eq 4 denotes the distance constraint that the distance from community *j* to shelter *i* is less than the maximum distance. Eq 5 ensures that one community can choose only one shelter. Eq 6 ensures that decision variables *B*_*ij*_ and *Y*_*i*_ can only equal 0 or 1.

### PSO algorithm

The original PSO algorithm is attributed to Eberhart and Kennedy [21] and was intended for use in simulating social behavior. It is characterized by a fast convergence, robustness and effectiveness and has thus become an important tool in solving complex optimization problems. The PSO algorithm has been applied in the fields of engineering design [22, 23], medical science [24], task allocation [25, 26] and social network [27].

In general, the standard PSO algorithm is used to solve continuous optimization problems and may be suitable for discrete optimization problems after modification. Modification of the PSO algorithm was essential in this work because the problem of the location and allocation of earthquake shelters is a typical discrete problem. There have been many studies on the discrete PSO (DPSO) algorithm, mainly on completing the discretion by discretizing in continuous space or calculating in discrete space. The discretizing PSO in continuous space (DPSOCS) algorithm has been developed more than the algorithm for calculating in discrete space (DPSODS). Kennedy and Eberhart [28] proposed a binary version to discretize the PSO by defining the position of a particle with binary 0–1 and by defining the velocity as the probability of the particle’s value being equal to 1. Although many methods have been proposed to modify this DPSO [29, 30], it is still very complex as a result of the process of encoding and decoding. Rounding the number of positions of particles is another important method [31]. This method only converts the positions of particles from real number space into integer space without changing any part of the standard equation for the calculation of the PSO. To use the PSO in discrete space, the calculation mechanism is always redefined. Clerc [32] solved the ‘traveling salesman problem’ by a switching mechanism to replace the PSO calculation. Pan *et al*. [33] and Hu *et al*. proposed [17] a new position update method by using a permutation representation in the PSO rather than the standard PSO update method. Although these methods are all useful in given situations, the DPSOCS is simpler, faster and more mature. Therefore in this work, we used the rounding method to handle position.

### Location and allocation of shelter

To smoothly apply the PSO algorithm to the problem of the location and allocation of shelters, we defined the position of the particle as *PS = (ps*_*1*_, *ps*_*2*_,…, *ps*_*j*_, *… ps*_*M*_ *)*, which represents the site selection assignment of the shelter. *ps*_*j*_ is the number of dimensions of the *j*th community, namely, the number of candidate shelters belonging to community *j*. *M* represents the total number of dimensions, namely, the total number of communities. Originally, *ps*_*j*_ = (1, 2, … *i*, … *N*); however, the *j*th community cannot select all the candidate shelters because of the distance constraint. Therefore, the candidate shelters of each community should be redefined; the indexes of the candidate shelters are not consecutive as a result of the distance constraint.

The shortest distance from each community to the candidate shelters are calculated using the Dijkstra algorithm. We assumed here that the people in a community behave as a group when evacuating and that the speed of different groups is different due to the different proportions of children and elderly people in the group. Thus, the maximum distance from the community to the shelter can be defined according to Eq 10:

$$D_{j}=t_{j}\times v_{j}$$ (10)

Assuming that each person will evacuate to the shelter within 60 min (*t*_*j*_ = 3600 s), the maximum distance of each community from the shelter can be obtained according to Eq 10. Thus, the covered matrix of communities can also be calculated as follows:

$$Covered(i,j)=\left\{ \begin{array}{l} 1\;if\;\mathrm{distance}(i,j)\leq D(j)\\ 0\;if\;\mathrm{distance}(i,j)>D(j) \end{array}\right.$$ (11)

The final candidate shelters of all communities will be obtained by combining the covered matrix and initial candidate shelters.

It is obvious that the number of final candidate shelters is consecutive and therefore needs to be renumbered. Initially, assuming that the number of candidate shelters the *j*th community can select is *N*, and that the serial number of candidate shelters the *j*th community can select initially is defined as *Number(j) =* (1, 2, *… i*, *… N*) (*j = 1*, *2*, *… M*), then *Newnum(j)* is the final number of the *j*th community, *Selenumber(j)* is the serial number of shelters for community *j* that satisfy the distance constraint, and *Newnumber(j) =* (1, 2, *… t*, *… Newnum(j)*) is the new serial number of shelters under the distance constraint. The rule of renumbering is as follows:

If

*covered(i*,*j)* = 1, then *Newnum(j)* = *Newnum(j)*+1

*Newnumber(j)* = (1, 2, …, *t*, … *Newnum(j)*)

**Fig 1:**
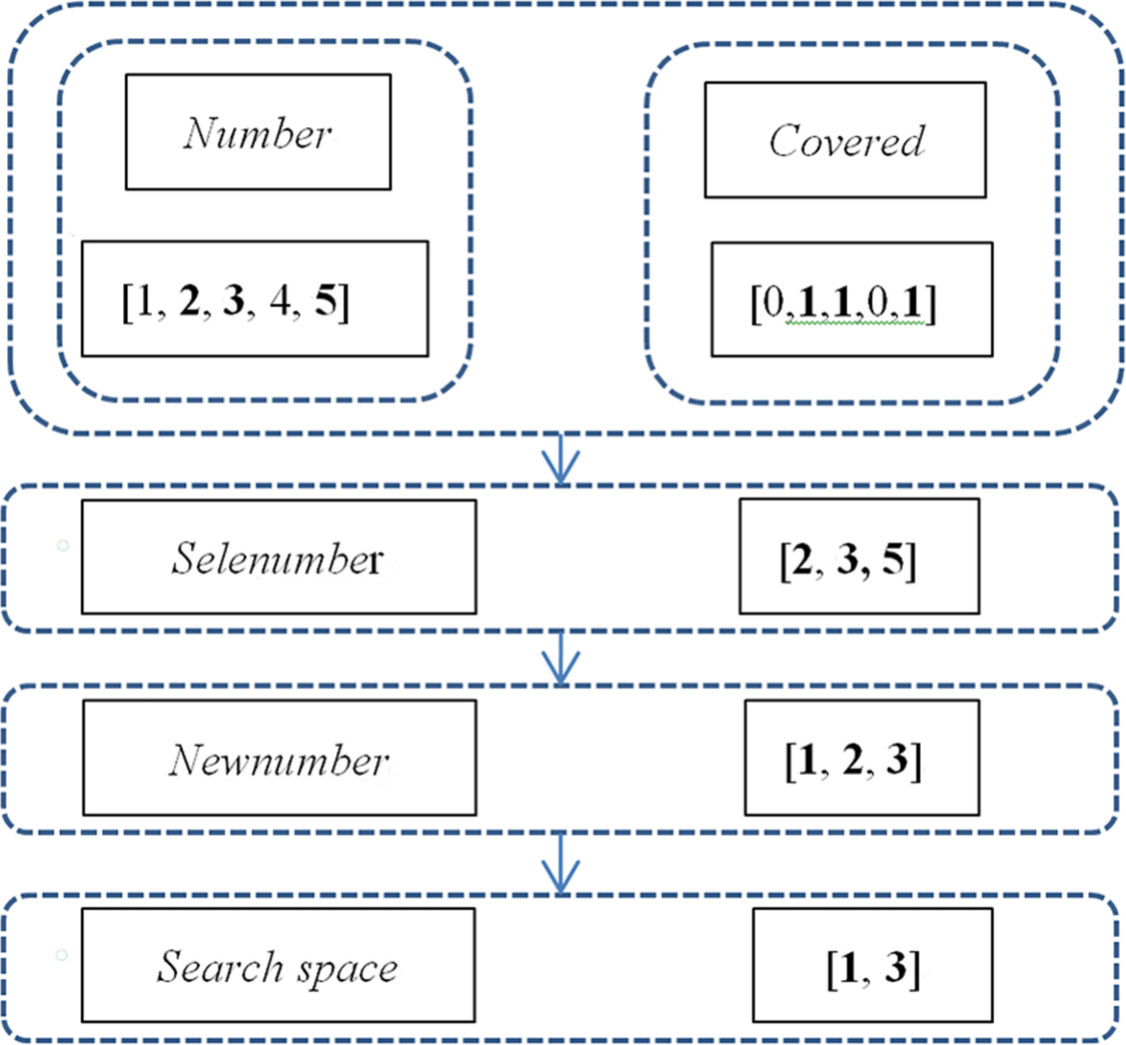
Conversion process from original serial number to new original serial number and search space for every particle.

Thus, *Newnumber(j)* can correspond to a unique original serial number *Nnumber(j)*. For example, community 1 has five candidate shelters numbered 1, 2, 3, 4 and 5. People of community 1 can arrive at shelters 2, 3 and 5 in 60 min, so the covered matrix of community 1 is covered (1, 5) = [0, 1, 1, 0, 1]. *Newnum(1) =* 3 *and Newnumber(1) =* [1,2,3]. The corresponding relationship is showed in Fig 1.

### Position update method

As the major part of the PSO, the update method is generally modified to improve the efficiency of solving complex problems. Shi and Eberhart [34] introduced an inertia weight into the original PSO algorithm to balance both the global search and the local search. Clerc and Kennedy [35] added a constriction factor into the PSO algorithm to ensure the convergence of the algorithm and to cancel the constraint of velocity. Besides that, the topology structure of PSO algorithm also affects the solving process. In this work, to effectively solve the problem, the constriction factor PSO with a rounding method is modified by applying the von Neumann structure in the former loops to explore all space and adding global structure in the later loops to exploit. The particle’s position can be updated as follows:

$$v_{i}\left(k+1\right)=\varphi \times \left(v_{i}\left(k\right)+c_{1}rand_{1}\times \left(pbest_{i}-p_{i}\left(k\right)\right)+c_{2}rand_{2}\times \left(lbest_{i}-p_{i}\left(k\right)\right)\right)$$ (12)

$$v_{i}\left(k+1\right)=\varphi \times \left(v_{i}\left(k\right)+c_{1}rand_{1}\times \left(pbest_{i}-p_{i}\left(k\right)\right)+c_{2}rand_{2}\times \left(gbest_{i}-p_{i}\left(k\right)\right)\right)$$ (13)

$$\varphi =\frac{2}{\left|2-c-\sqrt{c^{2}-4c}\right|},\;c=c_{1}+c_{2},\;c>4$$ (14)

$$p_{i}(k+1)=round(p_{i}(k)+v_{i}(k+1))$$ (15)

where *p(k) =* (*p*_1_ *(k)*, *p*_2_ *(k)*, *… p*_*i*_ *(k)*, *… p*_I_ *(k)*) represents the particle’s position in the *k*th generation (*k =* 1, 2, … *K*), where *K* is the maximum index of iteration; *v* (*k*) = (*v*_*1*_(*k*),*v*_*2*_(*k*), *… v*_*i*_(*k*) *… v*_*I*_(*k*)) is the velocity of the particle swarm; *I* is the dimension of a particle; *pbest*_*i*_ denotes individual optimum, *lbest*_*i*_ and *gbest*_*i*_ denote neighbor optimum and global optimum by using the von Neumann structure and global structure respectively; *c*_1_and *c*_2_ are learning factors; *rand*_1_ and *rand*_2_ are random numbers between 0 and 1; and *φ* is the constriction factor in the calculation mechanism in Eq 14.

Eq 12 and Eq 13 of the velocity update include three parts as follows:

1. *v*_*i*_ *(k)* is the current velocity inherited from the previous velocity
2. *c*_*1*_ × *rand*_*1*_ × (*pbest*_*i*_ − *p*_*i*_(*k*)) is ‘thinking by itself’, the particle’s cognition
3. in Eq 12, *c*_*2*_ × *rand*_*2*_ × (*lbest*_*i*_ − *p*_*i*_(*k*)): collaboration and information sharing among the neighbor particles; in Eq 13, *c*_*2*_ × *rand*_*2*_ × (*gbest*_*i*_ − *p*_*i*_(*k*)): collaboration and information sharing among the global particles

Eq 15 denotes the particle’s update mechanism with a rounding method. Although the constriction factor PSO can control the fly speed of the particle efficiently and enhance the local searching ability, it is easily trapped in the local optimum. SA can find the best solution by imitating the solids’ annealing procedure, thus avoiding trapping in the individual optimum and local optimum by accepting the worse solutions in certain probability [36, 37]. Combining PSO and SA, which can improve the ability of the algorithm to find better solutions, has been used as a local search for each particle’s individual optimum and local optimum [17].

### Multi-objective problems with constraints

To solve the problem of multi-objectives with constraints, a Pareto strategy and feasibility-based rule [22] were introduced to the PSO. Instead of calculating the average side-length of cuboid of *i*th solution in NSGA-II [38], the area which can present the dense of *i*th solution is calculated to obtain the local optimum and global optimum in this study. The solution with the largest area, namely, the least dense part in local non-inferior solutions and global non-dominated solutions, are selected as the local optimum and global optimum respectively.

**Fig 2:**
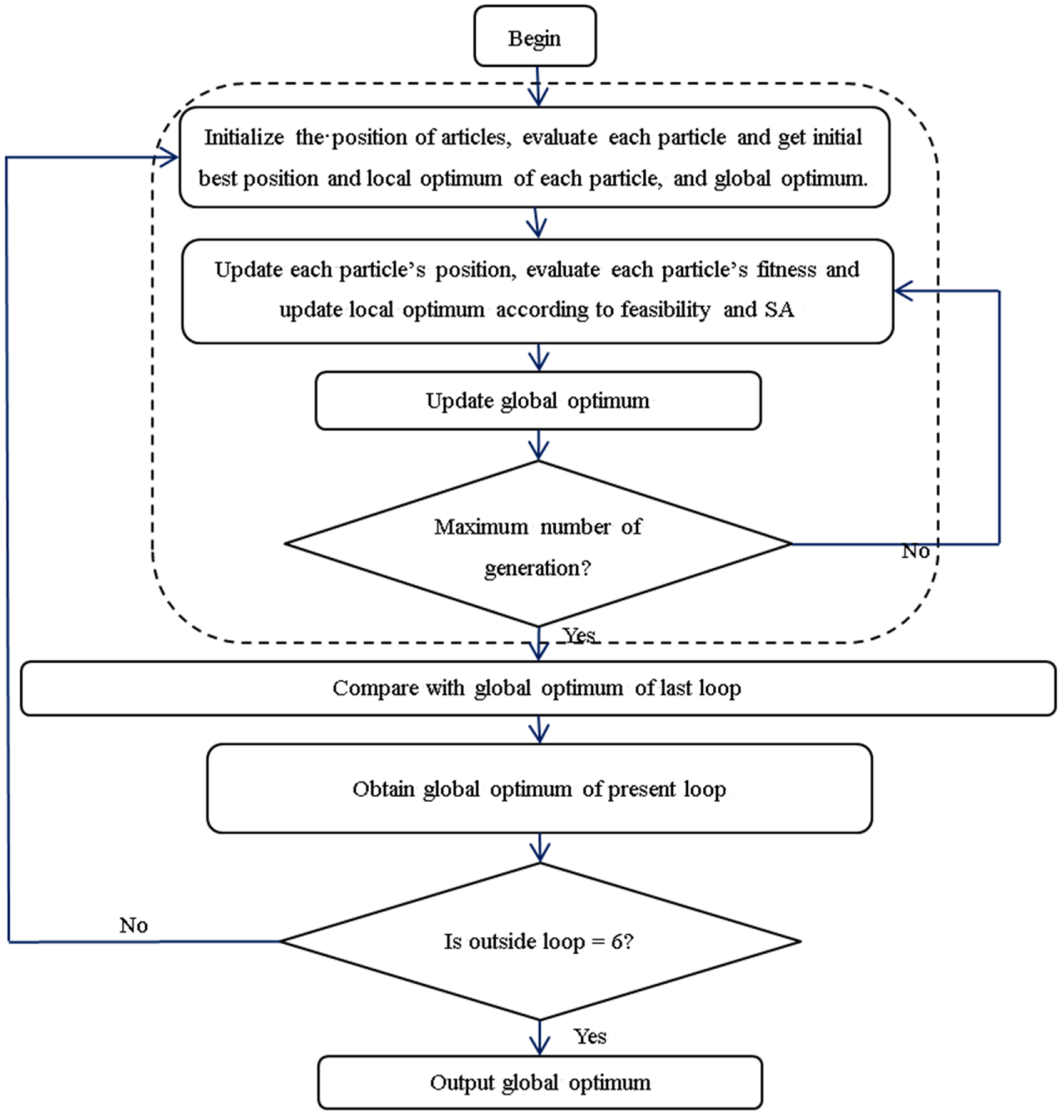
Flow diagram of modified PSO algorithm.

Considering the randomness of the PSO and the priority of objectives in selecting non-dominated solutions, an outside loop was set in this study. By comparing the solution of every inside loop with the solution of the last loop, the solution of the new loop was obtained until the maximum outside loop. The flow of the modified PSO algorithm is shown in Fig 2.

## Case study

### Study area

Located in a zone with a high risk of earthquakes, Beijing was the first city in China to build disaster emergency shelters. Chaoyang district, a central district in Beijing with a population of 3,500,000, has built six earthquake disaster shelters with the capacity to accommodate 500,000 evacuees [39].

### Data

#### Candidate shelter data

Open spaces, such as green spaces, playgrounds and parks, with slopes <20° and more than 500 m from earthquake faults were selected as shelters. The data can be digitized according to the Beijing municipality map. This assumes that the capacity of each evacuee in a shelter should be at least 1 m^2^.

#### Number of evacuees

The intensity of an earthquake and the damage to buildings greatly affects the number of evacuees. To make the research more practical, three scenarios were considered in this study: (1) scenario A (S_A_)–all people need to evacuate; (2) scenario B (S_B_)–earthquake of magnitude 8.0 in Sanhe-Pinggu; and (3) scenario C (S_C_)–magnitude 6.75 earthquake of magnitude 6.75 in Juyongguan.

According to the empirical equation of the seismic intensity attenuation model [40] and the fragility curve, the collapse rates of buildings for S_B_ and S_C_ were calculated to be 33.83% and 14.76%, respectively. Thus, the number of evacuees in each community was calculated according to Eq 9.

#### Evacuation route

The best route for each community to the shelter was calculated using the Dijkstra algorithm with the objective of maximizing the mean width of the road and minimizing the total length of the route and the total number of nodes in the route.

## Results and Analysis

The huge number of community and candidate shelter in Chaoyang District makes it difficult and time consuming to find the optimal solution with the developed model. Therefore, the solution that is near to the optimum and can be quickly obtained is generally regarded as the optimal solution. In this study, to find the solution, the maximum number of generation in every outside loop is set as 20,000 by considering the effectiveness and timeliness.

Learning factors *c*_1_ and *c*_2_ greatly influence the result, *c*_1_ = 2.05, *c*_2_ = 2.05 and *c*_1_ = 2.8, *c*_2_ = 1.3 are often used in current researches. This study selects learning factors of *c*_1_ = 2.8, *c*_2_ = 1.3 by analyzing the results of these two different learning factor groups without outside loop.

As a result of the complexity of this problem, and the influence of the sorting principle of variables (time and area), increasing numbers of generations might not give a better solution and therefore, to increase the diversity of particles and the effectiveness of the algorithm, and satisfy the demand of all variables, the outside loops were introduced. The candidate shelters of each community are sorted by evacuation time in the first two loops, are sorted by candidate shelter areas in the third and fourth loops, and are kept the initial positions in the last two loops. The global optimum of each outside loop was compared with the result of the last loop and the new result of the current outside loop was obtained. To obtain better solution, the final solutions are improved by combining the evacuation time.

The initial temperature and annealing rate were set to 1,000,000 and 0.96, respectively, according to the research of Hu *et al*. [17] and the parameters in the algorithm were set as in Table 1.

**Table 1:** Values of parameters used.

Parameter	Value (with outside loop)
Maximal outside loop	6
Maximal number of generation	20,000
Population size	100 (10*10)
Learning factor *c*_1_	2.8
Learning factor *c*_2_	1.3
Initial temperature	100,000
Annealing rate	0.96
Minimal temperature	0.01

**Fig 3:**
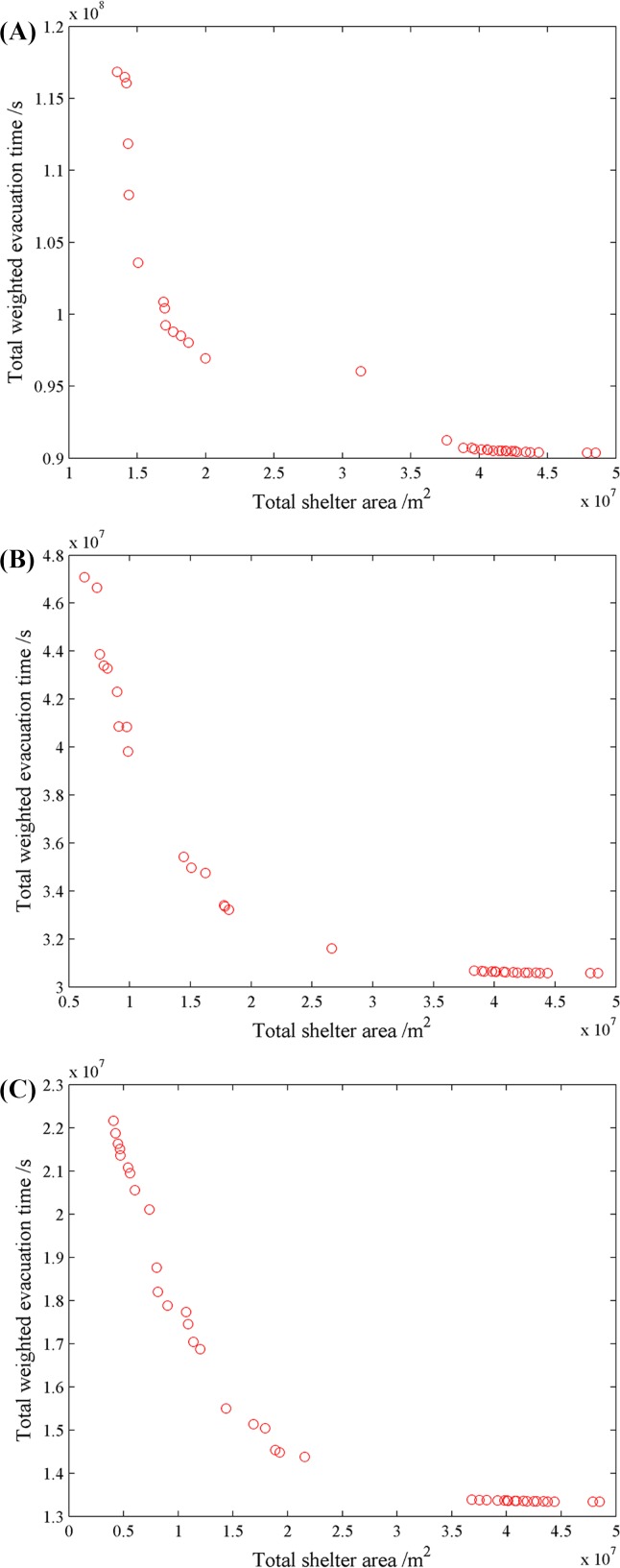
Results of different scenarios. (A) is the result of pareto-optimum solutions for S_A_, (B) is the result of pareto-optimum solutions for S_B_, and (C) is the result of pareto-optimum solutions for S_C_.

**Fig 4:**
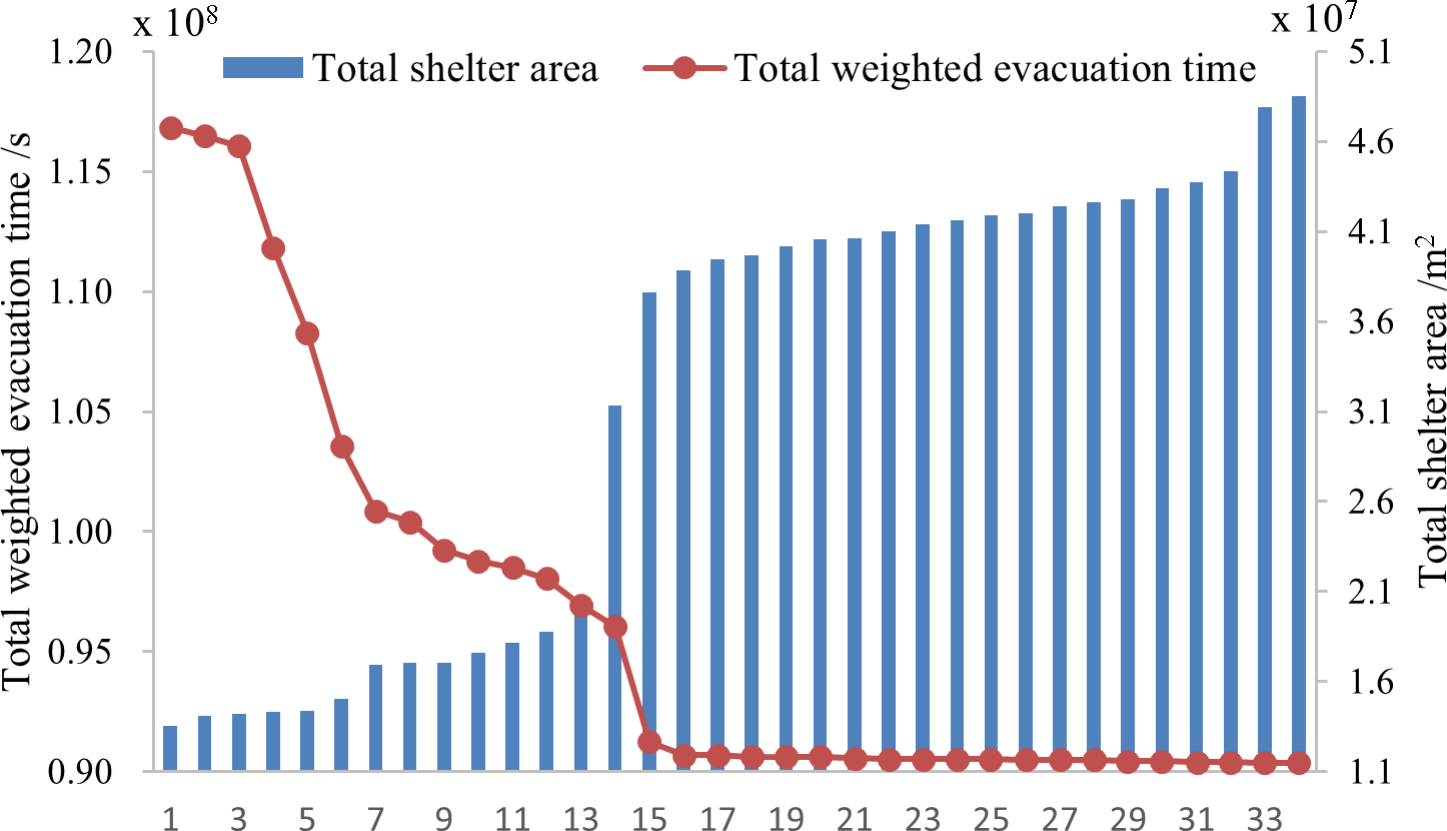
Pareto-optimal solutions for S_A_ in the last generation of the last outside loop.

**Fig 5:**
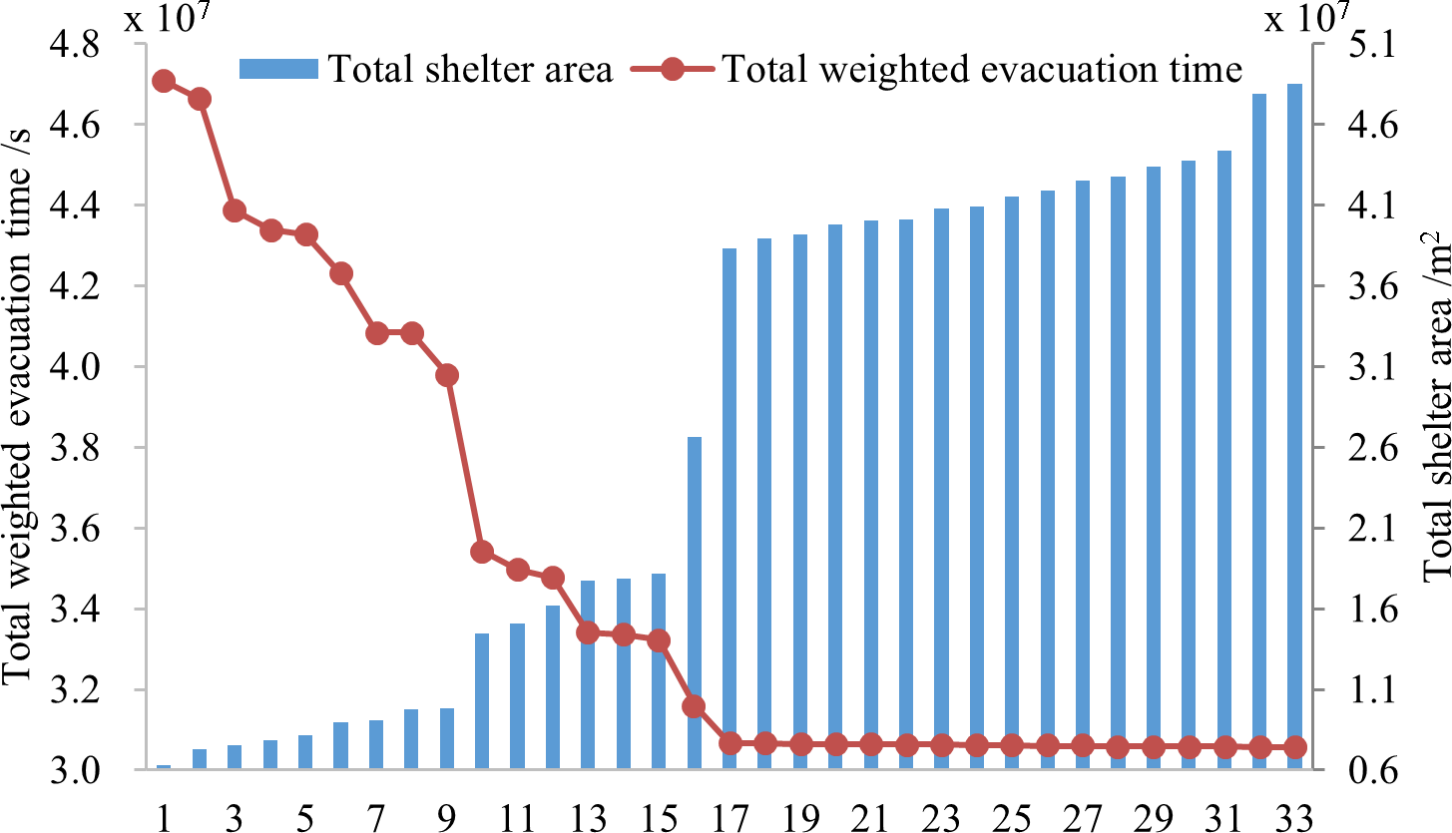
Pareto-optimal solutions for S_B_ in the last generation of the last outside loop.

**Fig 6:**
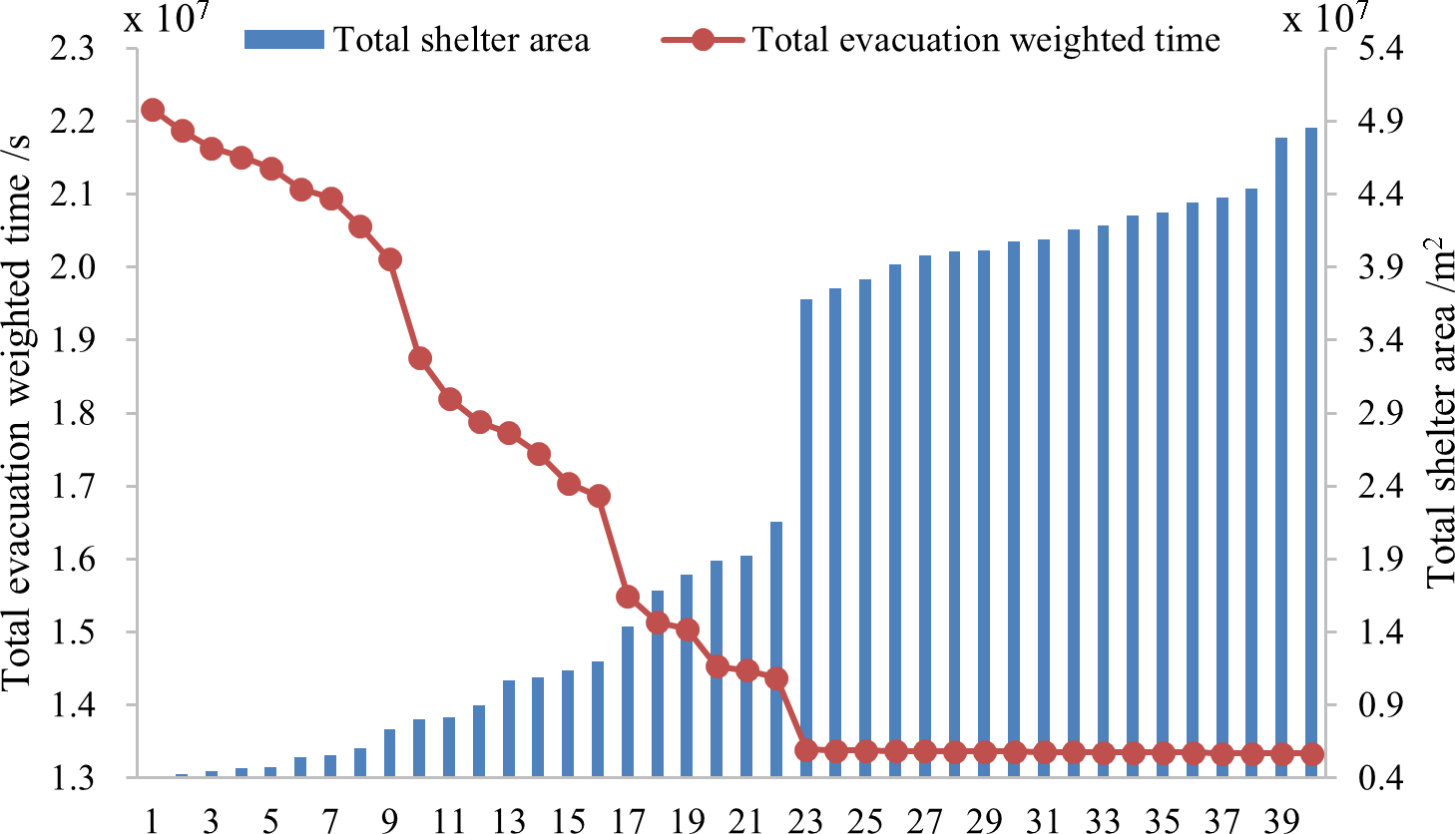
Pareto-optimal solutions for S_C_ in the last generation of the last outside loop.

Fig 3 illustrates the final results and Figs 4–6 show pareto-optimum solutions for S_A_, S_B_ and S_C_, respectively. There are 34 schemes, 33 schemes and 40 schemes for S_A_, S_B_, and S_C_ respectively. Total weighted evacuation time was negatively related to the total shelter area. The total weighted evacuation time decreased with increasing total shelter area. This suggests that the government should invest more in building shelters if a lower loss of life is desired. Decision-makers could select an appropriate scheme based on the financial conditions, the probabilities of different earthquakes and their own preferences.

**Fig 7:**
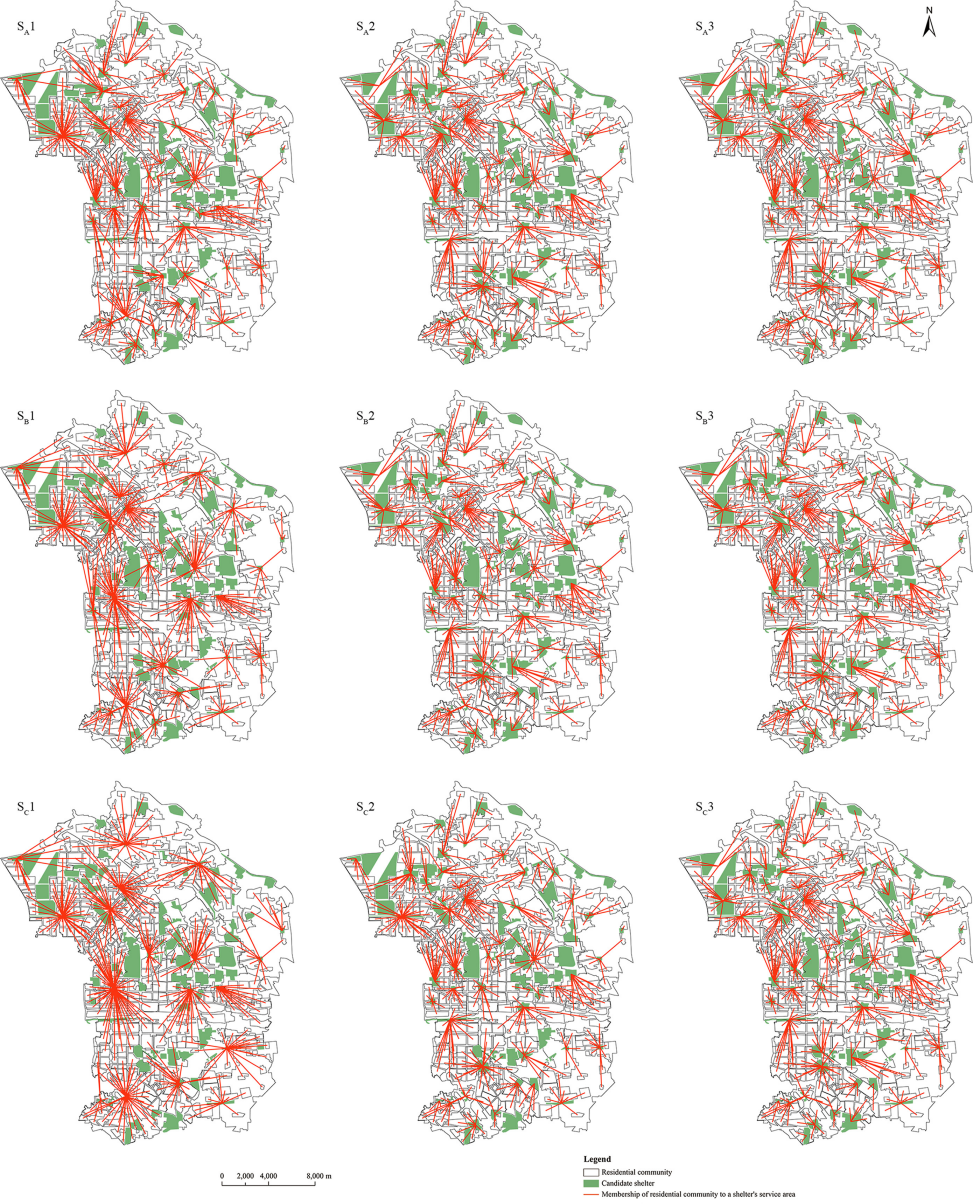
Schemes of earthquake shelter location and districting planning in three scenarios. (S_A_1), (S_B_1), and (S_C_1) are schemes with the minimal shelter area in scenarios S_A_, S_B_, and S_C_ respectively; (S_A_2), (S_B_2), and (S_C_2) are schemes with the median shelter area in scenarios S_A_, S_B_, and S_C_ respectively; (S_A_3), (S_B_3), and (S_C_3) are schemes with the maximal shelter area in scenarios S_A_, S_B_, and S_C_ respectively.

For each scenario, there are three schemes of earthquake shelter location and allocation planning which are displayed in Fig 7, which are selected from the Pareto-optimal solutions with minimal, median and maximal shelter areas. The communities and its shelter are connected with the red line. Some lines intersect because of route width’s influence on evacuation time. Shorter linear distance but narrower route makes evacuation time longer. Thus, the community will select the route with longer linear distance but shorter evacuation time. However, shorter evacuation time means more shelters, namely, the construction cost will also be increased. It is obvious that the number of shelters need to be opened vary in different scenarios. It is at least to open 43, 28 and 19 shelters in S_A_, S_B,_ and S_C_ respectively. To obtain the shortest evacuation time, the three scenarios need 65 numbers of shelters. The minimum average weighted evacuation time of these three scenarios is 25.65. The maximum average weighted evacuation time of S_A_, S_B_ and S_C_ are 33.16, 39.50 and 42.61 respectively. The evacuation population is 100%, 33.83% and 14.76% of the total population in S_A_, S_B_ and S_C_ respectively. A shelter can serve more than one community, and the people in a community can only evacuate to one shelter, thus, people in some communities should be allocated to the shelter that need more evacuation time to obtain the smallest area of shelters. It is shown in Table 2 that the maximum weighted average evacuation time of S_A_, S_B_, S_C_ is 33.16, 39.50 and 42.61 respectively. In the reality, the government must consider both people’s benefit (evacuation time) and their own capacity and resource (to build more shelters) when making the disaster shelter evacuation plan.

**Table 2:** Analysis of the results in scenario S_A_, S_B_ and S_C_.

	Minimum number of shelter	Maximum number of shelter	Minimum weighted average evacuation time (s)	Maximum weighted average evacuation time (s)
Scenario S_A_	43	65	25.65	33.16
Scenario S_B_	28	65	25.65	39.50
Scenario S_C_	19	65	25.65	42.61

## Conclusion

This study has introduced a multi-objective earthquake shelter allocation model by considering different evacuation scenarios using the Chaoyang district of Beijing as a case study. The PSO optimum algorithm was first modified by adding an outside loop and combing it with the SA algorithm and then used to solve the problem.

The results showed that the PSO algorithm with an outside loop was efficient in avoiding premature solutions and in finding more optimum solutions. It also showed that increasing the cost of construction can increase the profit according to the ratio of decreased evacuation time to increased shelter area. Increasing the shelter area can induce a large decrease in the total weighted evacuation time from scheme 1 to scheme 9 in S_A_, from scheme 1 to scheme 9 in S_B_, and from scheme 1 to scheme 19 in S_C_ while the effectiveness become less from scheme 10 in S_A_ and S_B_ and scheme 20 in S_C_, especially for the last 19 schemes of S_A_, the last 17 schemes of S_B_ and the last 18 schemes of S_C_, the evacuation time changes rarely. If funding were not a limitation, then the final schemes of each scenario were the best solutions; if funding was a limitation, then the earlier schemes were more reasonable.

The developed model, with its objectives of minimizing the area of shelters and the travel time, combining the capacity constraint and the distance constraint, is more practical than existing shelter location models. In this model, the speed parameter, which was obtained using the population weight of people of different ages, was considered in calculating the total weighted evacuation time, which makes the model more realistic. Three evacuation scenarios were considered to estimate the number of people who needed evacuate. Rounding the DPSO in continuous space was used in this study due to its simplicity. The outside loop was introduced to find a more optimum solution by solving the issues of the randomness of optimum algorithm and the complexity of the multi-objective model.

However, there is still scope to improve the model and to make it more practical. For example, as a result of the different unit costs of land, a unit cost can be added into the model for the land parameter to minimize the construction cost of the shelters. The evacuation speed could be optimized by integrating the evacuation behavior of residents and the condition of road collapse. PSO algorithm should be improved to solve the problem with the abundance data. Compared with the DPSOCS model, DPSODS can save more calculation space, but there are still many problems with it. Most DPSODS models are proposed by altering the PSO update mechanism according to the particular problem, so it is not applicable to other problems. Therefore, an effective DPSODS still needs be developed and applied in the location and allocation problem.
